# Retraction: Berberine Chloride Mediates Its Anti-Leishmanial Activity via Differential Regulation of the Mitogen Activated Protein Kinase Pathway in Macrophages

**DOI:** 10.1371/journal.pone.0341097

**Published:** 2026-01-20

**Authors:** The PLOS One Editors

After this article [1] was published, concerns were raised regarding results presented in Figs 2–4.

Specifically:

- The following lanes appear similar, despite representing different experimental results:
  - Fig 2F β-actin lane b, Fig 3A β-actin lane b, Fig 4A β-actin lane b, and Fig 4A IL-10 lane a
  - Fig 2F β-actin lane c, Fig 3A β-actin lane c, Fig 4A β-actin lane c, Fig 2F β-actin lane d, Fig 3A β-actin lane d, and Fig 4A β-actin lane d
  - Fig 2F β-actin lanes e-f, Fig 3A β-actin lanes e-f, Fig 4A β-actin lanes e-f, and Fig 4A IL-10 lanes b-c
- There appear to be vertical discontinuities in the following left and right gel images:
  - Fig 2F iNOS
  - Fig 3A IL-12p40
  - Fig 4A IL-10

Corresponding author MC stated that the left β-actin panels are the same image in Figs 2F, 3A, and 4A, and that the right β-actin panels are the same image in Figs 2F, 3A, and 4A. They stated that the target genes iNOS, IL-12p40, and IL-10 were analyzed under the same experimental conditions, using the same set of cDNA samples and the same PCR plate. They also stated that the original data underlying these results are no longer available. The authors did not respond to editorial request for comment regarding the above similarities between IL-10 and β-actin, or β-actin from different experimental conditions.

In light of the above unresolved concerns that question the reliability of the reported results, the *PLOS One* Editors retract this article.

PS, AS, AM, and MC agreed with the retraction. SB and SM either did not respond directly or could not be reached.
